# Association between Breakfast Skipping and the Metabolic Syndrome: The Korea National Health and Nutrition Examination Survey, 2017

**DOI:** 10.3390/medicina56080396

**Published:** 2020-08-07

**Authors:** JaeHun Jung, A-Sol Kim, Hae-Jin Ko, Hye-In Choi, Hee-Eun Hong

**Affiliations:** 1 Department of Family Medicine, Kyungpook National University Hospital, Daegu 41944, Korea; zeuse0258@naver.com (J.J.); liveforme@knu.ac.kr (H.-J.K.); blbr20@naver.com (H.-I.C.); hhe8824@naver.com (H.-E.H.); 2 Department of Family Medicine, School of Medicine, Kyungpook National University, Daegu 41944, Korea; 3 Department of Family Medicine, Kyungpook National University Chilgok Hospital, Daegu 41404, Korea

**Keywords:** skipping breakfast, metabolic syndrome, Korea National Health and Nutrition Examination Survey, intermittent fasting

## Abstract

*Background and Objectives:* Recently, the prevalence of metabolic syndrome in Korea has increased rapidly. Current knowledge reflects the importance of dietary control in relation to the metabolic syndrome. The objective of this study was to evaluate the influence of skipping breakfast on the metabolic syndrome. *Materials and Methods:* We conducted a cross-sectional study using data from the Korea National Health and Nutrition Examination Survey 2017 for the second year. A total of 3864 adults aged 20 to 64 were included in the study. We stratified the study population into three groups, based on breakfast patterns: the regular group, irregular group, and skipping group. Multiple logistic regression analysis was used to analyze the association between skipping breakfast and the presence of metabolic syndrome. *Results:* We noted an increase in the proportion of metabolic syndrome cases as follows: skipping group (3.3%), irregular group (5.4%), and regular group (8.5%) (*p* < 0.001). The multivariate-adjusted odds ratios of metabolic syndrome in the skipping and irregular groups compared with the regular group were 0.68 (95% CI; 0.35 to 1.35) and 0.81 (95% CI; 0.51 to 1.28), respectively. In the 40–65-year-old age group, which had a high prevalence of metabolic syndrome, the multivariate-adjusted odds ratios of metabolic syndrome in the skipping group compared with regular group were 0.78 (95%CI, 0.39 to 1.62). *Conclusions:* There was no significant correlation between skipping breakfast and risk factors of metabolic syndrome (after adjusting for risk factors), but a tendency of skipping breakfast to lower the risk of metabolic syndrome was observed. A rationale for these results is proposed through the association between skipping breakfast and intermittent fasting.

## 1. Introduction

Metabolic syndrome is a group of metabolic disorders characterized by abdominal obesity, hypertriglyceridemia, low blood high-density lipoprotein (HDL) cholesterol levels, high blood pressure, and insulin resistance [1]. The prevalence of metabolic syndrome is gradually increasing worldwide [2].

The prevalence of metabolic syndrome in South Korea was 19.6% in 2013 and 21% in 2015 [3], and it is known as a common disease. The incidence of metabolic syndrome is increasing, and in Asians, the morbidity of metabolic diseases is alarming [4]. Hence, the prevention and management of metabolic syndrome is fundamental. The pathophysiology of metabolic syndrome is known to be a combination of genetic, metabolic, and environmental factors [5]. Modifiable risk factors include lack of physical activity, weight gain, excessive alcohol consumption, and poor dietary behavior [6]. Among these, eating habits are very important in the prevention and management of metabolic syndrome [5].

Of the previous studies on the association between metabolic syndrome and eating habits, most were conducted with a focus on different classes of nutrients. Studies have shown that the consumption of carbohydrates and sodium is a major cause of increased risk of metabolic syndrome [7]. In addition, nuts, fruits, and vegetables reduce the risk of metabolic syndrome when eaten for breakfast [8]. In a study into the association between meal frequency and obesity, the authors found that body mass index decreased as the number of meals increased [9], whereas other studies reported that the total calorie intake increased and body fat increased as the number of meals increased [10]. Therefore, the relationship between the number of meals and metabolic syndrome is still controversial.

According to a survey of 446 office workers in Seoul, South Korea, most of the respondents (88.8%) knew the importance of breakfast, but only 33.2% of them were eating breakfast every day [11]. Among the three meals, 71% of people did not have breakfast, 15% did not have lunch, and 12% did not have dinner—the highest percentage of people did not eat breakfast [12]. According to the Korea National Health and Nutrition Examination Survey, the rate of skipped breakfast was 22.4% for men and 21.3% for women in 2010 and 29.5% for men and 25.7% for women in 2017, showing an increasing trend.

The proportion of breakfast-skippers is on the rise. However, prior studies on the relationship between breakfast and metabolic syndrome have been conducted only in people in a specific region or in a hospital. In addition, research has focused only on the relationship between breakfast-skipping and the intake of individual nutrients, which is insufficient. Therefore, this study aimed to provide meaningful information for the prevention and management of metabolic syndrome by analyzing the relationship between skipping breakfast and metabolic syndrome through the National Health and Nutrition Survey.

## 2. Materials and Methods

### 2.1. Study Population

This study was conducted on adults, aged 20 to 64, who participated in the National Health and Nutrition Survey for the 2nd year (2017). The National Health and Nutrition Examination was a statutory survey conducted for the purpose of calculating basic statistics on the health and nutrition of people under the National Health Promotion Act and was approved by the Institutional Review Board of the Korea Centers for Disease Control and Prevention. Indicators used for the diagnosis of metabolic syndrome are extracted from smoking, drinking, physical activity, and dietary survey data. Statistical analysis was performed by classifying people into groups: the skipping group, irregular group, and regular group, based on dietary variables. The exclusion criteria of this study were as follows: pregnant cases, those who are not provided with true weights, and subjects for whom a diagnosis of metabolic syndrome could not be made due to insufficient responses. A total of 3864 people (1643 men and 2221 women) satisfying the above criteria were selected as final subjects.

### 2.2. Anthropometric Measurements and Blood Biochemical Indices

In the data provided, the subject’s height, weight, body mass index, waist circumference, systolic and diastolic blood pressure, serum total cholesterol, serum triglyceride, serum HDL cholesterol, and fasting blood glucose were used. Height and weight were measured with the participants wearing light clothing and no shoes. Waist circumference was measured at the narrowest point between the upper iliac crest and the lowest rib after normal expiration. Blood pressure was measured by averaging three recordings taken in the morning after at least 10 min of rest in a sitting position. Blood pressure was measured twice, using a mercury sphygmomanometer, in the sitting position after at least 10 min of rest. Laboratory samples were obtained after 12 h fasting.

### 2.3. Diagnosis of Metabolic Syndrome

In this study, the diagnostic criteria for metabolic syndrome used were those outlined by the National Cholesterol Education Program Adult Treatment Panel III (NCEP ATP III) guideline [13]. Furthermore, the Asia-Pacific obesity treatment guidelines were applied to abdominal obesity, considering the difference in waist circumference between races [14]. A diagnosis was posed when three or more criteria listed below were met.

- Abdominal obesity: waist circumference ≥90 cm (male) and ≥80 cm (female)
- Triglycerides ≥150 mg/dL
- High density lipoprotein cholesterol (HDL): <40 mg/dL for men, <50 mg/dL for women
- Fasting blood glucose ≥100 mg/dL, insulin injection to treat diabetes, or taking oral hypoglycemic drugs
- Blood pressure: systolic blood pressure/diastolic blood pressure ≥130/85 mmHg or taking anti-hypertensive drugs

### 2.4. Classification According to the Breakfast Pattern

For classification according to the breakfast pattern, we used interview data from the Korea National Health and Nutrition Survey. The questionnaire concerned the frequency of breakfast for one week in the last year.

- Skipping group: 0 times/week
- Irregular group: 1–4 times/week
- Regular group: 5–7 times/week

### 2.5. Measurement of Variables

Parameters such as sex, age, smoking, drinking, and physical activity were used in the health survey as screening variables. Current smokers were defined as those who smoked more than five packs of cigarettes (100 cigarettes) in their lifetime, and still smoke. The average amount of alcohol consumed per week was seven or more glasses for men and five or more glasses for women, and those who drank more than twice a week in this manner were classified as heavy drinkers. Regular physical activity was defined as follows: (1) medium intensity physical activity of more than 2 h 30 min a week, high-intensity physical activity over 1 h 15 min, or mixing medium intensity and high intensity physical activity during physical exercise. Body mass index (BMI) was calculated as body weight/(height in meters)^2^.

### 2.6. Statistical Analysis

The National Health and Nutrition Survey was designed by the complex stratification system extraction method. It was thus analyzed by a complex sample design using stratification variables, colony variables, and weights. An independent t-test and chi-square test were conducted to analyze the differences between each group and the skipping breakfast group. Differences between breakfast-skipping patterns were compared using a generalized linear model. Multiple logistic regression analysis was used to analyze the association between skipping breakfast and metabolic syndrome parameters. Age, smoking, physical activity, drinking, and BMI were used as control variables, and were presented as the odds ratio (OR) with a 95% confidence interval (95% CI). All other statistical analyses were performed using IBM SPSS (Statistical Package for Social Sciences) for Windows version 25.0 (IBM SPSS Statistics, Armonk, NY, USA). Statistical significance was set at *p* < 0.05 in all analyses.

## 3. Results

### 3.1. General Characteristics of Study Population

The general characteristics of the study population were divided into three groups: the skipping group, irregular group, and regular group, as shown in Table 1. Sex (*p* < 0.001), age (*p* < 0.001), BMI (*p* = 0.03), drinking (*p* < 0.001), and smoking (*p* < 0.001) were the variables that showed a significant difference between the three groups. In the skipping group, there were more men than women, and in the regular group, more women than men. In the skipping group, people in their 20s were the most affected, but in the regular group, it was people in their 50s, meaning that men and young adults tended to skip breakfast more. In addition, the proportion of heavy drinkers and current smokers were highest in the skipping group.

The analysis of the differences in anthropometric measurements and blood tests between groups is displayed in Table 1. There were significant differences between the three groups (systolic blood pressure, height, weight, fasting blood glucose, and HbA1c). The systolic blood pressure was higher in the regular group (116.5 mmHg) than in the skipping group (113.8 mmHg), as was the fasting blood glucose (99.1 mg/dL and 96.1 mg/dL, respectively).

The prevalence of disease among the study groups is displayed in Table 1. Hypertension, dyslipidemia, diabetes, and myocardial infarction were the variables that showed a significant difference with respect to breakfast patterns. The prevalence of hypertension among all subjects was 11.4%, and was highest in the regular group (15.7%) compared to the skipping group (5.8%) and the irregular group (6.8%). The overall prevalence of dyslipidemia in the study population was 9.4%, and the prevalence of dyslipidemia was higher in the regular group (12.3%) compared to the skipping group (6.0%). In the case of diabetes, the overall prevalence was 4.1%, and the regular group (5.9%) had a higher prevalence rate than the skipping group (2.8%).

### 3.2. Analysis of Relationship between Breakfast Pattern and Metabolic Syndrome Components

When analyzing the differences in metabolic syndrome components amongst the three breakfast pattern groups, high blood pressure (*p* < 0.001), impaired fasting glucose (*p* < 0.001), and low HDL cholesterol (*p* < 0.001) were found to be significant. In the case of abdominal obesity and elevated triglycerides, there was no significant association (Table 2). The overall rate of diagnosis of metabolic syndrome according to NCEP ATP III is 6.6%. The proportion of metabolic syndrome was least in the skipping group (4.7%), compared to the irregular group (6.1%), and the regular group (10.5%). In the case of high blood pressure and impaired fasting glucose, the number increases as we move across the fasting group, irregular group, and regular group.

### 3.3. Risk of Metabolic Syndrome Related to Breakfast Patterns

The comparison of the skipping and irregular groups with the regular group is displayed in Table 3 as ORs. In the univariate analysis, the OR of the irregular group was 0.61 and the OR of the skipping group was 0.37 (*p* = 0.006, *p* < 0.001, respectively). In a multivariate analysis performed by adjusting sex, BMI, age, drinking, smoking, and physical activity, the OR of the irregular group was 0.81, and that of the skipping group was 0.68, though it was not statistically significant. (*p* = 0.354, *p* = 0.271, respectively).

The analysis of the risk factors of metabolic syndrome across different age groups of the study population is displayed in Table 4 and Table 5. In the 20–40-year-old group, the univariate analysis showed that the OR of the irregular group was 0.91 and that of the skipping group was 0.56, compared to the regular group. In multivariate analysis with adjustment of risk factors, the OR of the irregular group was 0.86 and that of the skipping group was 0.45. The ORs decreased, but this difference was not statistically significant. (*p* = 0.766, *p* = 0.296, respectively) In the 40–65-year-old group, the univariate analysis showed that the OR of the irregular group was 0.86 and 0.61 for the skipping group compared to the regular group (*p* = 0.435, *p* = 0.049, respectively) In the case of multivariate analysis with the control variables adjusted, the OR of the irregular group was 0.79 and that of the skipping group was 0.78, though this difference was not statistically significant.

**Figure 1:**
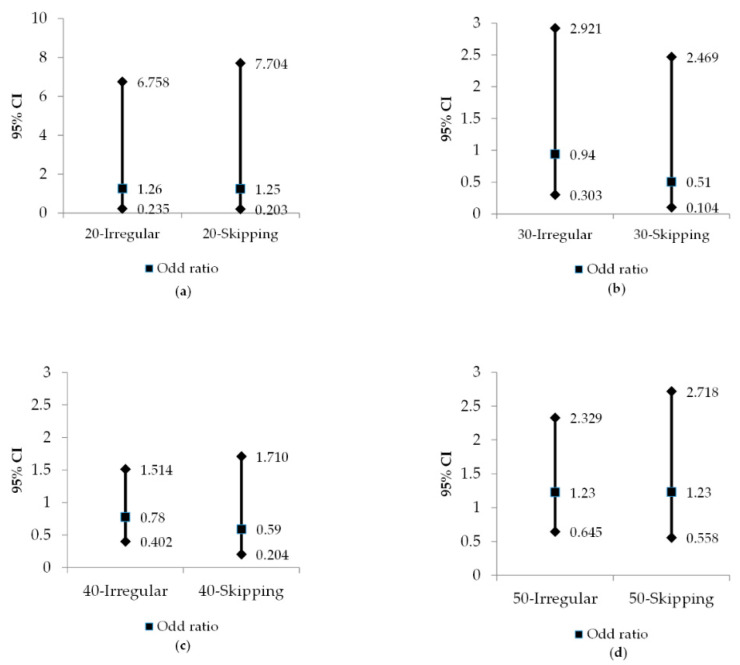
Odds ratio (OR) and 95% confidence intervals (CIs) for the metabolic syndrome according to age and breakfast pattern. (**a**) 20 ≤ age < 30, breakfast pattern; (**b**) 30 ≤ age < 40, breakfast pattern; (**c**) 40 ≤ age < 50, breakfast pattern; (**d**) 50 ≤ age < 64, breakfast pattern; Irregular: have breakfast 1~4 times per week; Skipping: have breakfast 0 times per week.

The results of multivariate logistic analysis across different age groups are illustrated in Figure 1. In those aged in their 20s and 50s, the OR of metabolic syndrome was one or higher in both the irregular group and the skipping group, compared to the regular group, but it was not significant.

## 4. Discussion

This study is a cross-sectional study that analyzes the association between skipping breakfast and the metabolic syndrome in Korean adults using the National Health and Nutrition Examination Survey. In the group eating breakfast regularly, there were more women, but there were more men in the group skipping breakfast. The rate of regular breakfast with respect to age groups was higher in the 40–50 middle-aged group than in the 20–30-year-old group. This shows that there are differences in breakfast habits between generations. In previous studies, the rate of skipping breakfast was higher in men than in women, and the rate increased with age, which was consistent with this study. One study suggested that the main reason for the skipping breakfast was the lack of time to prepare and eat food [15]. In addition, in the study of breakfast energy intake, men had 19.5% of their estimated daily energy needs and women had 23.7%, showing higher breakfast energy in women [16]. Therefore, the proportion of breakfast-eating in women is higher than in men, and the rate of skipping breakfast decreases as age increases.

When the subjects were divided into the three breakfast pattern groups and the average values of blood pressure, anthropometric measurements, and serum components were compared, there were significant differences observed for systolic blood pressure, fasting blood glucose, and HbA1c. The irregular breakfast group had the lowest values, whereas the regular group had the highest (Table 1). On the other hand, in a previous study of the skipping breakfast group, the basal metabolic rate decreased. This causes overeating in the fasting state and the blood sugar rapidly increases, thereby raising the waist circumference, fasting blood sugar and insulin secretion, and total cholesterol [17]. In addition, a systemic review of the association of skipping breakfast with cardiometabolic risk factors reported that people who skip breakfast are at high risk of becoming overweight or obese [18]. All these risk factors lead to cardiovascular disease, contrary to our study.

The prevalence of chronic diseases (hypertension, dyslipidemia, and diabetes) was the lowest in the skipping group. Furthermore, the prevalence of metabolic syndrome was lower in the skipping group than in the regular group. The prevalence of metabolic syndrome in all subjects was 8.5%. It shows a difference from the prevalence rate of South Korea in 2015 [3]. The reason for this difference was that the elderly (aged 65 and older) were excluded from the study. Concerning the components of the metabolic syndrome, the regular group had high rates of high blood pressure, high blood sugar, and low HDL cholesterol. Previous studies show that smoking rates are high in breakfast skippers, and there were high rates of drinking and low rates of exercise in breakfast-eating women [19]. Therefore, risk was analyzed by adjusting for sex, BMI, age, smoking, drinking, and physical activity affecting metabolic syndrome. As a result, in the skipping group, the OR of metabolic syndrome was 0.68, compared to that of the regular group, but there was no statistical significance. For people in their 40s or older, who had a high prevalence of metabolic syndrome, the skipping group had a lower relative risk of metabolic syndrome than the regular group (after adjusting for risk factors); the odds ratio was 0.78, but there was no statistical significance.

Generally, it is known that skipping breakfast is considered a poor eating habit and that it can cause obesity and diabetes [20]. However, most of these studies were done in Western countries. In addition, according to meta-analysis of research from Asian and Pacific regions, although there was a positive association between skipping breakfast and prevalence of overweight and obesity, there were limitations due to the method of collecting data by questionnaire and memory recall [21]. Moreover, there are still limitations to suggesting a link between breakfast and metabolic disorder in adults from the Asia-Pacific region.

Several studies that analyzed breakfast and metabolic risks have suggested that skipping breakfast increases total cholesterol and LDL cholesterol, reduces postprandial insulin sensitivity [22], and increases blood pressure [19]. However, in another study of those who regularly ate breakfast, the triglycerides, fasting blood glucose, systolic and diastolic blood pressure were significantly high in the regular breakfast groups (more than 300 kcal for breakfast), and HDL cholesterol was low [23]. In the regular breakfast group, carbohydrate intake was found to be higher than in the skipping breakfast group, and triglyceride level, a risk factor for metabolic syndrome, was significantly higher [24]. As such, the relationship between breakfast pattern and metabolic syndrome is inconsistent. Hence, it is not only the eating of breakfast that counts, but also the amount of food and the ratio of nutrients consumed.

As a result of this study, there was no significant difference in abdominal obesity found between the regular breakfast group, the irregular group, and the skipping group. In a related prior study, it was found that skipping breakfast prevents feeling full when eating, [25], thereby causing obesity and overweight [20]. However, a recent cross-sectional study of women in their 20s in Japan showed that the total daily energy consumption of those who skip breakfast was lower than those who ate breakfast [26]. In addition, a study of adults aged 30–59 years in Korea showed a lower total energy intake in the skipping breakfast group [24]. In addition, in a study in Japan on the relationship between skipping breakfast and metabolic syndrome, people who simply did not eat breakfast had no association with metabolic syndrome. Only those who did not eat breakfast after eating dinner late at night had a relationship with metabolic syndrome and obesity [27]. Therefore, it is difficult to conclude that skipping breakfast is a bad eating habit, because skipping breakfast is not always associated with abdominal obesity and metabolic syndrome.

Although there was no statistical significance in multivariate analyses, the reason the relative risk of metabolic syndrome may be lower in the skipping breakfast group can be found in intermittent fasting, an activity into which many studies have been conducted recently. Intermittent fasting means eating habits that repeatedly limit calories from 12 h to several days [28]. Research on intermittent fasting has been actively conducted, since prior studies in animals showed that calorie restriction can help reduce aging and prolong life by reducing free radical production [29]. Several rationales have been suggested on this subject.

Blood glucose, amino acids, and insulin secretion are reduced by intermittent fasting or calorie restriction. This inhibits the insulin-like growth factor 1(IGF-1) pathway and mammalian target of rapamycin (mTOR). As a result, responses such as stimulation of mitochondrial production at the cellular level, increased resistance to stress, regeneration of damaged cells, and increased cell life occur. Therefore, intermittent fasting or calorie restriction can help resist disease [30]. Fasting causes glucose depletion, resulting in a “metabolic switch” in which the energy source is converted to a ketone body, thus increasing the concentration of ketone bodies in serum. These ketone bodies express peroxisome proliferator-activated receptor γ coactivator 1α (PGC-1α), fibroblast growth factor 21 (FGF21), and nicotinamide adenine dinucleotide (NAD+), which all act to prevent cell aging [31].

The most widely studied methods of intermittent fasting are fasting every other day (alternate day fasting), fasting two days a week (periodic fasting), and time restricted feeding (8–12 h feeding, 12–16 h fasting) [27]. Among the subjects in this study, those who skipped breakfast would be considered time restricted feeding.

Intermittent fasting in an animal study reduced basal blood glucose and basal insulin, and increased insulin sensitivity, improving risk factors for obesity and cardiovascular disease [32]. Intermittent fasting has been reported to be effective for weight loss in obese people [33]. In addition, a study showed that a 6-month caloric restriction improved cardiovascular risk factors, insulin sensitivity, and mitochondrial function [34]. Considering regularly skipping breakfast as one form of intermittent fasting, based on the results of the above studies, it can be suggested that regular breakfast-skipping can lead to regular and intermittent fasting, thereby preventing the risk of metabolic syndrome.

There are a few limitations encountered in this study. First, it was cross-sectional study. Therefore, it is difficult to present the causal relationship clearly, because the temporal sequential relationship between skipping breakfast and metabolic syndrome is unknown. Second, people who are diagnosed with diseases such as hypertension, diabetes, or dyslipidemia and who take medicine regularly may tend to have regular breakfasts, compared to those who do not have the diseases. It cannot be excluded that this has influenced the results of this study. However, since the definition of having a metabolic syndrome component includes not only those who have been diagnosed with the disease, but also those who meet the reference values in the test, the results of this study can be considered valuable. Third, data were collected using the self-written questionnaire surveys completed by subjects in the National Health and Nutrition Survey. There may be some bias because investigation with questionnaires requires the recall method. Finally, the calories and nutritional components of breakfast were not considered, and no was distinction made between breakfast and snack intake.

Despite these limitations, this study adjusts the risk factors associated with metabolic disease. In our analysis of the risk of metabolic syndrome by age group, we tried to measure independently the relationship between skipping breakfast and metabolic syndrome.

## 5. Conclusions

In conclusion, when the risk factors of metabolic syndrome were adjusted, there was no significant correlation between skipping breakfast and risk factors of metabolic syndrome, although we found the tendency to skip breakfast to be associated with a low incidence of metabolic syndrome. The rationale for these results can be explained by the association between skipping breakfast and intermittent fasting. In the future, we recommend that large scale prospective studies on breakfast habits and the risk of metabolic syndrome be conducted.

## Figures and Tables

**Table 1:** General characteristics of study population.

			Regular (N = 2237)	Irregular (N = 1016)	Skipping (N = 611)	*p*-Value
		N (%) or Mean	N (%) or Mean(SE)	N (%) or Mean (SE)	N (%) or Mean (SE)	
Sex	Male	1643 (49.1)	911 (46.9)	423 (48.8)	309 (56.0)	<0.001 ^(1)^
	Female	2221 (50.9)	1326 (53.1)	593 (51.2)	302 (44.0)	
Age (years)	20–29	548 (19.3)	156 (9.6)	225 (28.0)	167 (34.4)	<0.001
	30–39	784 (22.0)	311 (15.3)	290 (28.8)	183 (31.2)	
	40–49	964 (24.7)	551 (26.5)	270 (24.1)	143 (20.0)	
	50–59	1051 (24.5)	766 (33.1)	193 (16.5)	92 (11.7)	
	60–64	517 (9.6)	453 (15.5)	38 (2.7)	26 (2.8)	
BMI (kg/m2)	<18.5	151 (4.1)	68 (3.0)	49 (5.2)	34 (5.7)	0.03
	18.5–23.0	1543 (39.3)	875 (39.3)	433 (40.1)	235 (38.1)	
	>23.0	2158 (56.6)	1287 (57.7)	532 (54.6)	339 (56.2)	
Alcohol	Non-drinker	526 (13.1)	68 (15.6)	117 (10.9)	58 (9.3)	<0.001
	Social drinker	2145 (60.4)	875 (60.3)	586 (61.8)	336 (58.6)	
	Heavy drinker	904 (26.5)	1287 (24.1)	257 (27.3)	185 (32.2)	
Smoking	Non-smoker	2360 (57.5)	1432 (60.4)	618 (58.1)	310 (48.0)	<0.001
	Ex-smoker	746 (20.2)	459 (22.1)	178 (18.2)	109 (17.6)	
	Current smoker	758 (22.3)	346 (17.5)	220 (23.7)	192 (34.4)	
Exercise	None	1929 (50.9)	1115 (51.0)	497 (49.8)	317 (52.4)	0.7
	Regular	1737 (49.1)	1008 (49.0)	469 (50.2)	260 (47.6)	
HTN	No	3351 (88.6)	1844 (84.3)	942 (93.2)	565 (94.2)	<0.001
	Yes	513 (11.4)	393 (15.7)	74 (6.8)	46 (5.8)	
Dyslipidemia	No	3452 (90.6)	1932 (87.7)	952 (94.1)	568 (94.0)	<0.001
	Yes	412 (9.4)	305 (12.3)	64 (5.9)	43 (6.0)	
DM	No	3669 (95.9)	2088 (94.1)	992 (98.4)	589(97.2)	<0.001
	Yes	195 (4.1)	149 (5.9)	24 (1.6)	22 (2.8)	
Stroke	No	3832 (99.3)	2213 (99.0)	1011 (99.6)	608 (99.6)	0.141
	Yes	32 (0.7)	24 (1.0)	5 (0.4)	3 (0.4)	
MI	No	3847 (99.6)	2222 (99.4)	1014 (99.9)	611 (100.0)	0.003
	Yes	17 (0.4)	15 (0.6)	2 (0.1)	0 (0.0)	
Angina	No	3834 (99.4)	2213 (99.1)	1012 (99.6)	609 (99.8)	0.143
	Yes	30 (0.6)	24 (0.9)	4 (0.4)	2 (0.2)	
Systolic blood pressure (mmHg)		115.2	116.5 (0.4)	113.6 (0.6)	113.8 (0.7)	<0.001 ^(2)^
Diastolic blood pressure (mmHg)		76.4	76.7 (0.3)	76.6 (0.5)	76.4 (0.5)	0.74
Height (cm)		165.7	164.4 (0.3)	166.7 (0.4)	167.9 (0.5)	<0.001
Weight (kg)		65.96	64.8 (0.3)	66.7 (0.6)	68.3 (0.7)	<0.001
Waist circumstance (cm)		81.19	81.4 (0.3)	80.6 (0.5)	81.65 (0.6)	0.21
Fasting Blood Glucose (mg/dL)		97.8	99.1(0.5)	96.4 (1.0)	96.1 (0.9)	0.01
HbA1c		5.55	5.621 (0.0)	5.46 (0.0)	5.475 (0.0)	<0.001
Total Cholesterol (mg/dL)		194.88	194.52 (1.0)	195.95 (1.6)	194.28 (1.9)	0.61
Serum HDL-cholesterol (mg/dL)		51.91	51.66 (0.3)	52.79 (0.5)	51.28 (0.6)	0.4
Serum Triglycerides (mg/dL)		135.24	134.42 (3.0)	134.73 (5.2)	138.44 (6.2)	0.81

^(1)^*p* from χ2-test for categorical variables, ^(2)^ *p* from ANOVA for difference between means, SE: Standard error, BMI: Body mass index, HDL: High-density lipoprotein, HTN: Hypertension, DM: Diabetes mellitus, MI: Myocardial infarction.

**Table 2:** Prevalence of the subjects with risk values in each metabolic syndrome parameter.

		Total N (%)	Regular N (%)	Irregular N (%)	Skipping N (%)	*p*-Value
Metabolic syndrome						
	No	3537 (91.5)	2001 (89.5)	954 (93.9)	582 (95.3)	<0.001
	Yes	327 (8.5)	236 (10.5)	62 (6.1)	29 (4.7)	
Abdominal Obesity ^(1)^						
	No	2567 (66.8)	1451 (65.2)	705 (70.0)	411 (67.4)	0.306
	Yes	1277 (33.2)	776 (34.8)	302 (30.0)	199 (32.6)	
Elevated blood pressure ^(2)^						
	No	2610 (67.5)	1407 (62.9)	754 (74.2)	449 (73.5)	<0.001
	Yes	1254 (32.5)	830 (37.1)	262 (25.8)	162 (26.5)	
Impaired fasting glucose ^(3)^						
	No	2703 (70.0)	1491 (66.7)	752 (74.0)	460 (75.3)	<0.001
	Yes	1161 (30.0)	746 (33.3)	264 (26.0)	151 (24.7)	
Elevated triglycerides ^(4)^						
	No	2610 (67.5)	1464 (65.4)	730 (71.9)	416 (68.1)	0.172
	Yes	1254 (32.5)	773 (34.6)	286 (28.1)	195 (31.9)	
Reduced HDL-C ^(5)^						
	No	2568 (66.5)	1414 (63.2)	731 (71.9)	423 (69.2)	<0.001
	Yes	1296 (35.3)	823 (36.8)	285 (28.1)	188 (30.2)	

^(1)^ Abdominal obesity: waist circumference ≥90 cm in men, ≥80 cm in women, ^(2)^ Elevated blood pressure, Systolic blood pressure ≥130 mmHg or Diastolic blood pressure ≥ 85 mmHg or taking anti-hypertensive drugs, ^(3)^ Impaired fasting glucose, serum fasting glucose ≥100 mg/dL or insulin injection to treat diabetes or taking oral hypoglycemic drugs, ^(4)^ Elevated triglycerides, serum triglycerides ≥ 150 mg/dL, ^(5)^ Reduced HDL-C, HDL-cholesterol <40 mg/dL in men, <50 mg/dL in women, Metabolic syndrome: the presence of 3 or more of the 5.

**Table 3:** Odds ratio (OR) and 95% confidence intervals (CIs) of prevalence of metabolic syndrome according to breakfast pattern.

Metabolic Syndrome	Breakfast	Odd Ratio	95% CI		*p*-Value
Model 1	Regular	1			
	Irregular	0.61	0.435	0.867	0.006
	Skipping	0.37	0.234	0.584	<0.001
Model 2	Regular	1			
	Irregular	0.54	0.369	0.791	0.002
	Skipping	0.37	0.207	0.656	0.001
Model 3	Regular	1			
	Irregular	0.84	0.561	1.25	0.384
	Skipping	0.66	0.356	1.204	0.172
Model 4	Regular	1			
	Irregular	0.81	0.509	1.275	0.354
	Skipping	0.68	0.345	1.351	0.271

Model 1: unadjusted, Model 2: adjusted for sex and BMI, Model 3: adjusted for sex, BMI, and age, Model 4: adjusted for sex, BMI, age, alcohol, smoking, and exercise.

**Table 4:** Age < 40 y, odds ratio (OR) and 95% confidence intervals (CIs) of metabolic syndrome.

Metabolic Syndrome	Breakfast	Odd Ratio	95% CI		*p*-Value
Model 1	Regular	1			
	Irregular	0.91	0.397	2.064	0.811
	Skipping	0.56	0.187	1.652	0.288
Model 2	Regular	1			
	Irregular	0.65	0.245	1.698	0.373
	Skipping	0.38	0.1	1.456	0.158
Model 3	Regular	1			
	Irregular	0.71	0.266	1.902	0.494
	Skipping	0.43	0.104	1.756	0.237
Model 4	Regular	1			
	Irregular	0.86	0.31	2.371	0.765
	Skipping	0.45	0.1	2.025	0.296

Model 1: unadjusted, Model 2: adjusted for sex and BMI, Model 3: adjusted for sex, BMI, and age, Model 4: adjusted for sex, BMI, age, alcohol, smoking, and exercise.

**Table 5:** Age ≥ 40 y, odds ratio (OR) and 95% confidence intervals (CIs) of metabolic syndrome.

Metabolic Syndrome	Breakfast	Odd Ratio	95% CI		*p*-Value
Model 1	Regular	1			
	Irregular	0.86	0.595	1.251	0.435
	Skipping	0.61	0.373	0.999	0.049
Model 2	Regular	1			
	Irregular	0.73	0.473	1.133	0.16
	Skipping	0.65	0.366	1.168	0.15
Model 3	Regular	1			
	Irregular	0.87	0.557	1.362	0.542
	Skipping	0.74	0.401	1.377	0.343
Model 4	Regular	1			
	Irregular	0.79	0.473	1.324	0.371
	Skipping	0.78	0.385	1.616	0.515

Model 1: unadjusted, Model 2: adjusted for sex and BMI, Model 3: adjusted for sex, BMI, and age, Model 4: adjusted for sex, BMI, age, alcohol, smoking, and exercise.
